# Gonorrhœa of the Rectum

**Published:** 1873-08

**Authors:** 


Gonorrhœa of the Rectum.—I have had under my care three cases of undoubted gonorrhœa of the rectum. There was great heat and burning pain experienced, with a copious discharge of pure pus; the mucous membrane, as seen through the speculum, was intensely inflamed; the cases occurred in prostitutes. The cure was not difficult; lead lotion and opium was used in two cases, and answered very well; the third was cured by sulphate of zinc and warm water injections three times daily. In neither case was there any ulceration of the lining membrane of the bowel, nor did any thickening or contraction result; the inflammation did not appear to affect the submucous areolar tissue.—*Allighan, Diseases of Rectum*, 1873, *p.* 264. (*Clinic*, *Feb.*, 1873.)

